# F18-FDG-PET for recurrent differentiated thyroid cancer: a systematic meta-analysis

**DOI:** 10.1177/0284185115594645

**Published:** 2015-07-09

**Authors:** Torjan Haslerud, Katrin Brauckhoff, Lars Reisæter, Regina Küfner Lein, Achim Heinecke, Jan Erik Varhaug, Martin Biermann

**Affiliations:** 1 Nuclear Medicine/PET-Center, Department of Radiology, Haukeland University Hospital, Bergen, Norway; 2 Section for Endocrine Surgery, Haukeland University Hospital, Bergen, Norway; 3 Section for Oncological Imaging, Department of Radiology, Haukeland University Hospital, Bergen, Norway; 4 Medical and Dental Library, University of Bergen, Bergen/Norway; 5 Institute of Biostatistics and Clinical Research, University of Münster, Münster/Germany; 6 Department of Clinical Science, University of Bergen, Bergen/Norway; 7 Department of Clinical Medicine, University of Bergen, Bergen/Norway

**Keywords:** Head/neck, thyroid, neoplasms, PET-CT

## Abstract

**Background:**

Positron emission tomography (PET) with fluor-18-deoxy-glucose (FDG) is widely used for diagnosing recurrent or metastatic disease in patients with differentiated thyroid cancer (DTC).

**Purpose:**

To assess the diagnostic accuracy of FDG-PET for DTC in patients after ablative therapy.

**Material and Methods:**

A systematic search was conducted in Medline/PubMed, EMBASE, Cochrane Library, Web of Science, and Open Grey looking for all English-language original articles on the performance of FDG-PET in series of at least 20 patients with DTC having undergone ablative therapy including total thyroidectomy. Diagnostic performance measures were pooled using Reitsma’s bivariate model.

**Results:**

Thirty-four publications between 1996 and 2014 met the inclusion criteria. Pooled sensitivity and specificity were 79.4% (95% confidence interval [CI], 73.9–84.1) and 79.4% (95% CI, 71.2–85.4), respectively, with an area under the curve of 0.858.

**Conclusion:**

F18-FDG-PET is a useful method for detecting recurrent DTC in patients having undergone ablative therapy.

## Introduction

Differentiated thyroid cancer (DTC) is the most common malignant endocrine tumor. Although prognosis is generally favorable, with reported 5-year survival rates of 95% for women and 87% for men (1), some patients continue to experience adverse outcomes despite improvements in imaging and surgical technique (2,3).

Serum human thyroglobulin (hTg) is a reliable marker for persistent or recurrent disease after previous ablative therapy with total thyroidectomy with or without additional ablative radioiodine therapy (RIT). Routine ultrasound of the neck is often negative in these patients, as we recently demonstrated in a prospective cohort from our institution (2). Scanning with radioactive iodine, in particular after a therapeutic activity of iodine 131 (I-131), may reveal tumor lesions missed by conventional imaging (4). In two seminal papers in 1995 and 1996, Feine et al. demonstrated that positron emission tomography (PET) with fluor-18-deoxy-glucose (FDG) can detect tumor lesions that are missed by I-131-scintigraphy (5, 6). To explain their findings, they postulated a “flip-flop-phenomenon”, whereby highly differentiated thyroid cancer cells show iodine uptake due to the expression of sodium-iodide symporter (NIS) but no glucose uptake, while less differentiated cells that ceased to express NIS exhibit upregulated glucose and FDG uptake (7–9).

Since then F18-FDG-PET has become an important method in patients with DTC with suspected recurrent or persistent DTC, as is evidenced in many series published since the late 1990s. A systematic meta-analysis, published by Dong et al. in 2009, documented a pooled patient-based sensitivity of F18-FDG-PET of 83.5% and specificity of 84.3% (10). We recently published results from multimodal imaging including F18-FDG-PET from a prospective cohort of 51 patients (2) and wanted to compare our results with more current data from the literature.

## Material and Methods

We performed a systematic literature search for all English-language original articles addressing diagnostic performance of F18-FDG-PET in series of 20 patients or more with suspected or known recurrence after previous ablative therapy published from 1996 until 31 December 2014.

More specifically, our selection criteria were: (i) all patients had undergone previous ablative therapy including total thyroidectomy; (ii) patients were suspected of having recurrences and/or metastases or had risk factors such as a measurable or rising hTg or circulating hTg antibodies; (iii) patients underwent an FDG-PET or combined hybrid FDG-PET/computed tomography (CT) of the torso; (iv) reported study data were sufficient to calculate sensitivity and specificity for tumor detection; (v) histology, cytology, or follow-up were used as gold standard. Exclusion criteria were: article types other than original articles such as abstracts, letters, editorials, and comments. When data or subsets of data were presented in more than one article, the most recent article was chosen.

A systematic literature search was conducted on 17 February 2015 in five databases including Ovid MEDLINE(R)/PubMed (http://www.ncbi.nlm.nih.gov/pubmed; U.S. National Library of Medicine, Bethesda/MD), EMBASE (Ovid), Cochrane Library (Wiley), Web of Science (Thomson Reuters), and Open Grey (http://www.opengrey.eu) using standardized subject headings (MeSH, EMTREE) for thyroid cancer and FDG or PET as well as their free-text equivalents. The detailed search strategy can be obtained from the author.

All of 3625 hits were screened based on abstract and title by two authors (MB, TH), and then further analyzed based on the full manuscript. All relevant manuscripts were available in full text format. To assess the methodologic quality and the applicability of the included articles, the QUADAS-2 instrument was independently applied by the two authors (11). As suggested by the QUADAS-2 instrument, additional signaling questions were defined. Based on the follow-up results from our own cohort (2) we introduced an extra signaling question “Is the mean or median follow-up duration after imaging two years or more?” and we assumed a risk of bias regarding the time/flow domain when it was not (see Supplementary Materials 1 [online only] for further details on the study-specific signaling questions and scoring criteria).

All pertinent data from the included articles were registered in MDCake, a dedicated client-server database application developed by our group for data collection and management (12). The observers were blinded to each other when entering QUADAS-scores. Data were re-aggregated into a single SQL view for qualitative as for statistical analysis. Pooled diagnostic performance was estimated using R package *mada* based on Reitsma’s bivariate model (13,14). The application of univariate models for pooling sensitivities and specificities in diagnostic studies is no longer considered appropriate (15).

## Results

The literature search identified 34 studies with a total of 2639 patients meeting the inclusion criteria (6,16–48). Eleven studies reported on the diagnostic accuracy of single-modality PET, 17 on PET/CT, and six on both PET and PET/CT (“mixed”). The pertinent details of the 34 studies are presented in Supplementary Materials 2, Table (online only).

**Fig. 1.**
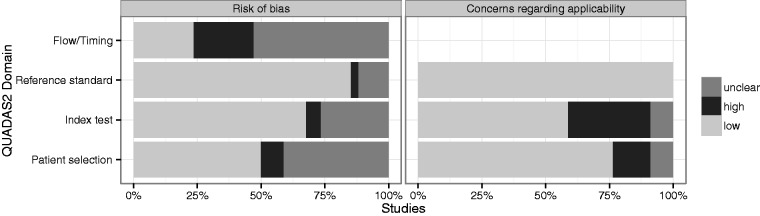
Results of QUADAS-2 scoring of the 34 component studies according to the four QUADAS-2 domains patient selection, index test, reference standard, and flow/timing (11).

*Methodological quality.* QUADAS-2 scores are summarized in Fig. 1. According to the QUADAS-2 standard, patient selection should be consecutive in a prospective study design (11). The precise mode of patient selection was however unclear in 14 (41%) of the studies and apparently biased in three (9%). The index test should be applied blinded to the outcomes or in a prospective manner. This was insufficiently documented in nine (26%) of the studies. Most frequently the reviewers had concerns regarding the timing and the flow of the studies: only in eight (24%) of the studies was the duration of post-imaging follow-up considered sufficient, while potential bias was found in 18 (53%) of the studies and high risk of bias in eight (24%).

**Fig. 2.**
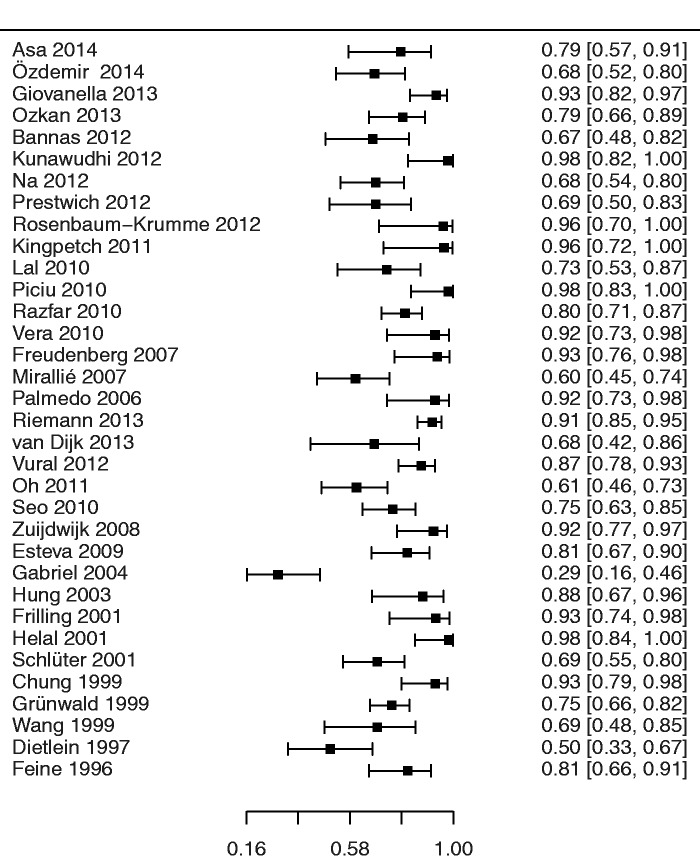
Forest plot of the diagnostic sensitivities of the 34 component studies with 95% confidence intervals (continuity corrected).

**Fig. 3.**
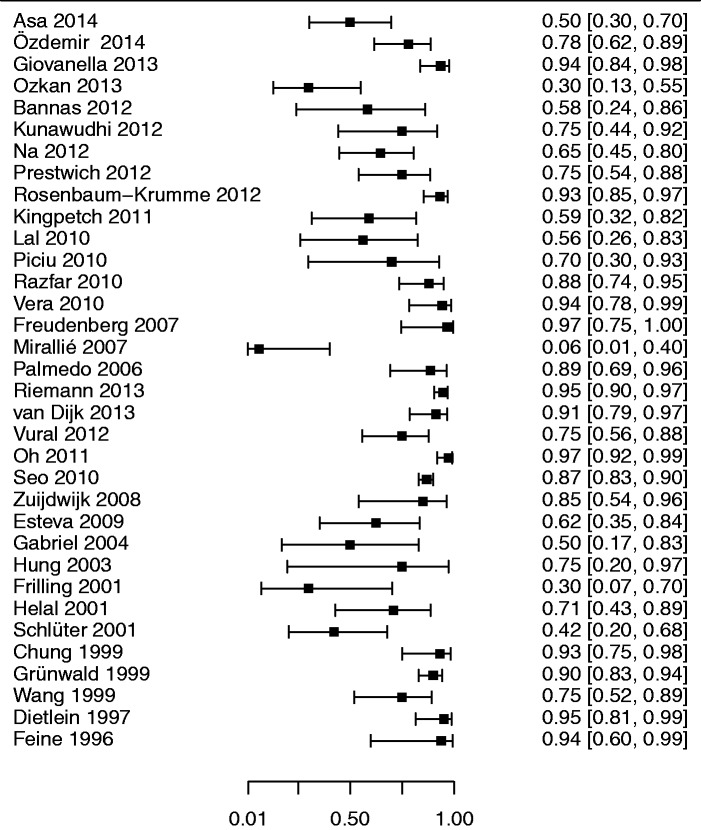
Forest plot of the diagnostic specificities of the 34 component studies with 95% confidence intervals (continuity corrected). The raw specificity in the study by Mirallié et al. was 0% (seven false positive FDG-PET studies among the seven patients without detectable disease) (37).

**Fig. 4.**
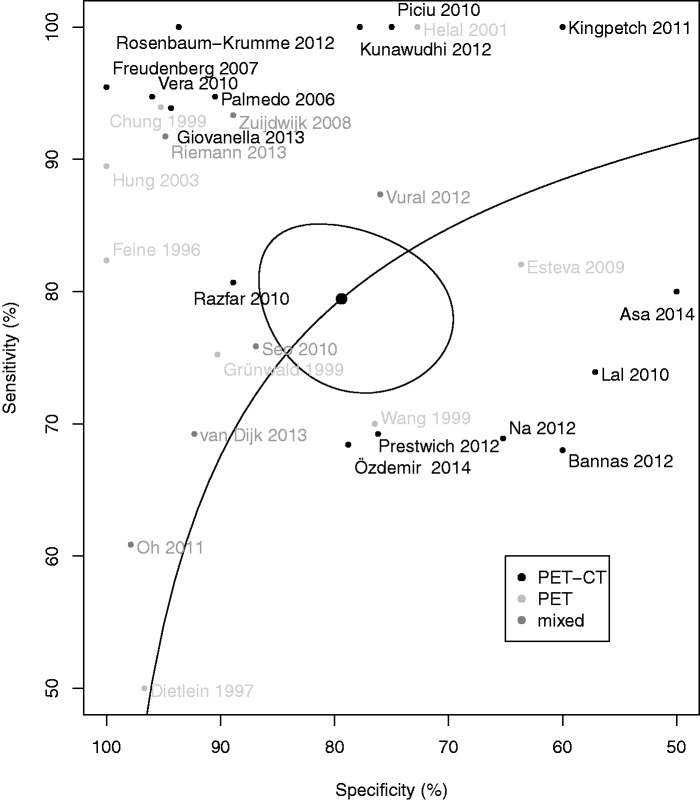
Summary receiver operating characteristics (SROC) curve of the 34 component studies. Pooled sensitivity/specificity over all studies is marked in the center of the plot with a surrounding 95% confidence ellipse. Sensitivity and specificity of the individual studies are plotted in ROC space. PET, studies using single-modality PET; PET-CT hybrid PET + CT; mixed, studies reporting on a mixture of PET and PET-CT; PET, studies using single-modality PET; PET-CT, hybrid PET + CT.

*Meta-analysis.* Forest plots of sensitivity and specificities in the component studies are presented in Figs. 2 and 3. Pooled sensitivity and specificity of all studies in relation to the reference standard were 79.4 % (95% confidence interval [CI], 73.9–84.1) and 79.4% (95% CI, 71.2–85.4), respectively, with an area under the curve of 0.858 (Fig. 4). In a subgroup analysis, PET-CT performed slightly better than single modality PET (Table 1), but the difference was not statistically significant.

**Table 1.** Pooled diagnostic performance of PET and PET/CT.

Type	Studies (*n*)	Patients (*n*)	Pooled sensitivity	Pooled specificity	AUC
PET	11	640	76.6% (62.8–86.4)	75.7% (59.5–86.8)	0.826
PET-CT	17	905	80.2% (73.5–85.6)	75.5% (62.8–85.0)	0.844
Mixed	6	1094	82.1% (69.4–90.2)	91.2% (82.8–95.7)	0.933
All	34	2639	79.4% (73.9–84.1)	79.4% (71.2–85.4)	0.858

Pooled diagnostic performance measures with 95% confidence intervals.

AUC, area under the summary receiver operating characteristics (SROC) curve; PET, studies using single-modality PET only; PET-CT hybrid PET + CT only; mixed, mixture of PET and PET-CT in the same study; PET-CT, hybrid PET + CT.

An AUC of 1 represents an ideal test while a non-discriminatory test will have an AUC of 0.5.

## Discussion

F18-FDG-PET is a useful tool for evaluating patients with suspected recurrence of DTC with a pooled sensitivity of 79.4% and specificity of 79.4% across all studies. These results compare well to the most recent systematic meta-analysis published in 2009, which reported a pooled patient-based sensitivity and specificity of 81% (74–86%) and 82% (73–88%) based on 17 studies using comparable statistical methodology (10).

The stable, rather than improved diagnostic performance over the past 5–6 years was surprising, given the latest developments in imaging technology such as high resolution PET-CT (Table 1). Although PET-CT performs better than single-modality PET in head-to-head comparisons (2,38), the effect did not reach statistical significance in the meta-analysis. Interestingly, pooled specificity was highest for “mixed” studies reporting on cohorts examined with either single-modality PET or PET-CT. We regard this as an outlier as the “mixed” studies did not differ in their QUADAS-2 scores from the PET-CT studies.

The apparently constant diagnostic performance of PET despite major advances in imaging technology can hardly be explained by the considerable heterogeneity of the individual studies uderlying the present meta-analysis, by publication bias or overoptimistic reporting (49,50). We suspect that this rather points to two methodical flaws common to most studies on our meta-analysis. The generally accepted definition of a “true positive” examination is the finding of at least one true positive lesion in the imaging study confirmed in a surgical specimen or a biopsy regardless of the number of possible false negative lesions that may have been overlooked. True positive and false negative lesions can, however, coexist in the same patient, and if overlooked in a thyroid cancer patient, may lead to avoidable repeat surgery (2). Similarly, false positive lesions, which may lead to unnecessary surgery, will not be scored in patients who have at least one true positive lesion. Thus patient-based sensitivity and specificity tend to be overoptimistic compared with lesion-based analysis. The second problem lies in the composite reference standard that is based on a combination of pathology, imaging, and clinical outcomes. As discussed in our recent publication, prophylactic surgery for DTC in the absence of imaging findings is considered unethical (2). Thus the only means of identifying false negative (overlooked) findings in an imaging study is careful patient follow-up. To a large extent, this follow-up relies on the same imaging techniques (e. g. ultrasound and PET) in which the false negative lesion was potentially overlooked. Thus the reference standard is not independent from the index test. An independent reference standard is, however, the fundamental assumption underlying diagnostic accuracy studies (11). A similar issue occurs if image-guided biopsy forms part of the composite standard: Only lesions that are seen can be biopsied, thus a biopsy will not guard against overlooked lesions. Thyroid cancer is, as a rule, a slow growing tumor. In our QUADAS-2 analysis, we therefore stipulated that the mean or median follow-up should be 2 years or more. This was based on our experience that, until now, four overlooked false negative lesions in our series were detected 0.9, 1.1, 1.1, and 3.8 years after the initial PET study (2). We therefore suggest that future diagnostic imaging studies in solid tumors employ both patient-based and lesion-based analysis, and follow-up of sufficient duration for the cancer type under study.

Our meta-analysis has the following limitations. First, we restricted our analysis to English language articles. Second, we excluded studies with fewer than 20 subjects. Meaningful estimates of sensitivity and specificity need a minimum number of cases with and without disease (51). However, the choice of the threshold was arbitrary given that disease prevalence varies between studies. Third, we abstained from conducting extensive subgroup analyses, such as on the role of TSH stimulation on the diagnostic performance of FDG-PET, the presence or absence of iodine uptake, or the correlation of serum hTg or hTg antibodies and true positive PET findings. Given the considerable heterogeneity between the component studies, we do not think a meta-analysis will provide reliable valid conclusions. Finally, we did not pool lesion-based sensitivities and specificities as there are were only five studies that presented lesion-based diagnostic performance data (Supplementary Table).

In conclusion, F18-FDG-PET continues to be a useful method for detecting recurrent thyroid cancer, with a pooled patient-based sensitivity of 79.4% and specificity of 79.4% across all studies.

## Supplementary Material

Supplementary material

## Supplementary Material

Supplementary material
