# Supplementary material for: F18-FDG-PET for recurrent differentiated thyroid cancer: a systematic meta-analysis
Source: Acta Radiol. 2015 Jul 9;57(10):1193–200. doi: 10.1177/0284185115594645 (PMC5015757; doi:10.1177/0284185115594645)
Supplement: Supplementary material [file BiermannSupplMat1.pdf]

Supplementary Materials 2: QUADAS 2 assessment

QUADAS is a tool for the Quality Assessment of studies of Diagnostic Accuracy included in Systematic reviews developed by Whiting et al. in 2003 (1). Based on experience with QUADAS, a revised version was issued in 2011 (2).

Under QUADAS 2, the “quality” of a given study in relation to the question under review is independently assessed in four domains: patient selection, index test, reference test, and flow/timing. All four domains include two independent judgments: “*Risk of bias*” and “*Applicability*”. Only the last domain “flow/timing” comprises a single judgment on “Risk of Bias”. All seven judgments are scored “Yes” or “No” or “unclear”. The “unclear” category is reserved for studies that reported insufficient information for the assessment.

To aid reproducible classification, the judgments are aided by signaling questions. These are specific to a given systematic review. Depending on the review question under study, standard signaling questions may be omitted or extra questions introduced (2). All articles included in a systematic metaanalysis are judged independently by two observers. The final scores are then allocated in a consensus meeting between the observers. We refer to the QUADAS-2 background document for further details (3).

In the following, we explain our definitions for quality assessment of the studies in our metaanalysis in terms of QUADAS-2 methodology.

Domain 1: Patient selection

The purpose of the signaling questions is to help score the QUADAS criterion “*Risk of bias*” as “low”, “high” or “unclear”. The following signaling questions are based on the QUADAS-2 recommendation that a diagnostic study should ideally enroll all consecutive, or at least a random sample of, eligible patients with suspected disease. The QUADAS criterion “*Applicability*” addresses concerns that the patients included in the diagnostic study may differ from the target population in the metaanalysis in terms of severity of the target condition, demographic features, presence of co-morbidity and other conditions (such as presence of hTg-antibodies), setting of the study or previous testing protocols (e. g. previous PET-imaging).

Signaling question 1: Was a consecutive or random sample of patients employed?

Signaling question 2: Was a case-control design avoided?

Signaling question 3: Did the study avoid inappropriate exclusions?

Judgment 1: Risk of bias: “low”, “high”, “unclear”

Judgment 2: Concerns regarding applicability: “low”, “high”, “unclear”

Domain 2: Index test

QUADAS-2 recommends that the index test be conducted in a uniform manner with observers blinded to the final diagnosis. The purpose of the signaling questions is to help score the QUADAS criterion “*Risk of bias*” as “low”, “high” or “unclear”. The QUADAS criterion “*Applicability*” addresses concerns that the index test in a given study may be conducted in a different manner than in the target population based on variations in test technology, execution or interpretation of the results. Based on the advances in scanner technology in recent years, we scored “Applicability” with “yes” for all studies employing PET-CT, “no” for all studies using single-modality PET only, and “unclear” for mixed cohorts employing both PET and PET-CT.

Signaling question 1: Were the index test results interpreted without the knowledge of the reference standard? – “Yes” implies that the observers in the study were blinded as to the final diagnosis in the study based on the gold standard. This will be the case in all prospective study designs.

Signaling question 2: If a threshold was used, was it pre-specified? - If standardized uptake value (SUV) was used for categorizing FDG-uptake as malignant it had to be specified in advance, not set a posteriori. If visual scoring (uptake higher than background) was used, we considered this equivalent to a pre-specified threshold.

Judgment 1: Risk of bias: "low", "high", "unclear"

Judgment 2: Concerns regarding applicability: "low", "high", "unclear"

Domain 3: Reference standard

QUADAS-2 recommends that the reference standard correctly identifies the target condition and that it is conducted in a consistent manner with observers blinded to the final diagnosis. The purpose of the signaling questions is to help score the QUADAS criterion "*Risk of bias*" as "low", "high" or "unclear". The QUADAS criterion "*Applicability*" addresses concerns that the target condition identified by the reference standard does not match the research question. We have chosen to score "Applicability" as "yes" when the diagnostic endpoint was presence or absence of morphologically identifiable tumor lesions, but "no" when the endpoint was total survival or a serological marker such as hTg (4).

Signaling question 1: Is the reference standard likely to identify the target condition? – Most diagnostic studies on DTC use a composite gold standard comprising histology of surgical specimens, cytology based on image-guided fine needle biopsies, and follow-up. Follow-up as part of the composite gold standard is unavoidable in thyroid cancer studies due to ethical concerns. Histology as sole gold standard would imply that all patients be operated, even those with no detectable disease on imaging (5). Also, some patients may have significant co-morbidity precluding surgery for tumor recurrence that is not life-threatening. We scored signaling question 1 as "Yes" when the composite standard include histology and/or cytology as well as clinical/imaging follow-up. We included an extra signaling questions on the quality of the clinical/imaging follow-up:

Signaling question 2: Was the clinical/imaging follow-up appropriate to identify the targeting condition? – This question addresses the intensity of the follow-up and the modalities chosen. At the very minimum we stipulate blood samples (TSH, hTg, Tg-antibodies or hTg-recovery) and cervical ultrasound, in case of lesions outside the neck repeat imaging (CT or PET-CT), otherwise the answer to the signaling question was "No".

Signaling question 3: Were the reference standard results interpreted without knowledge of the results of the index test? – This condition can be assumed to be met for histological and cytological specimens, but not for imaging follow-up as interpretation of follow-up images should always include comparison with previous imaging. We answered the question with "Yes" when more than 50 % of the classifications of the composite gold standard were based histology/cytology rather than follow-up.

Judgment 1: Risk of bias: "low", "high", "unclear"

Judgment 2: Concerns regarding applicability: "low", "high", "unclear"

Domain 4: Flow and timing

QUADAS-2 recommends that the index test and the reference standard be conducted in the same patient at the same time. The purpose of the signaling questions is to help score the QUADAS criterion "*Risk of bias*" as "low", "high" or "unclear".

Signaling question 1: Was there an appropriate interval between index test and reference standard? – Given that thyroid cancer is generally slow growing, we answered the question with "yes" when the mean/median time between PET-scanning and surgery and/or biopsy was 3 months or less.

Because of the important of follow-up as the "soft" component in the composite gold standard, we added an additional signaling question:

Signaling question 2: Was the duration of clinical/imaging follow-up appropriate? – Adequate follow-up is closely related to its duration. DTC is a slow-growing cancer. In our own cohort of 51 patients, we discovered cancer lesion that were overlooked on primary PET-imaging but later found on subsequent imaging/clinical follow-up in four patients after follow-up durations of 0.9, 1.1, 1.1, and 3.8 years (5). Based on these findings and our clinical experience, we stipulated a mean or median follow-up of at least two years in order to answer the signaling question with "yes".

Signaling question 3: Did all patients receive the same reference standard? **–** We answered the question with “yes” when the patients were subjected to the same composite gold standard.

Signaling question 4: Were all patients included in the analysis?

Judgment 1: Risk of bias: “low”, “high”, “unclear”. As a general rule, the risk of bias was assumed to be “low” with mean follow-up times of two years or more, “unclear” with follow-up times between 0.5 and 1.9 years or missing information on follow-up duration and “high” with documented follow-up times shorter than 0.5 years.

References

1. Whiting P, Rutjes AWS, Reitsma JB, et al. The development of QUADAS: a tool for the quality assessment of studies of diagnostic accuracy included in systematic reviews. BMC Med Res Methodol 2003;3:25.

2. Whiting PF, Rutjes AWS, Westwood ME, et al. QUADAS-2: a revised tool for the quality assessment of diagnostic accuracy studies. Ann Intern Med 2011;155:529–536.

3. QUADAS group. QUADAS-2: Background document [Internet]. 2014. .Available from: http://www.quadas.org

4. Durante C, Haddy N, Baudin E, et al. Long-term outcome of 444 patients with distant metastases from papillary and follicular thyroid carcinoma: benefits and limits of radioiodine therapy. J Clin Endocrinol Metab 2006;91:2892–2899.

5. Biermann M, Kråkenes J, Brauckhoff K, et al. Post-PET ultrasound improves specificity of 18F-FDG-PET for recurrent differentiated thyroid cancer while maintaining sensitivity. Acta Radiol 2015 (in press) doi:10.1177/0284185115574298
