# Supplementary material for: F18-FDG-PET for recurrent differentiated thyroid cancer: a systematic meta-analysis
Source: Acta Radiol. 2015 Jul 9;57(10):1193–200. doi: 10.1177/0284185115594645 (PMC5015757; doi:10.1177/0284185115594645)
Supplement: Supplementary material [file SRAD-2015-0249_Suppl_Mat2.pdf]

Supplementary Materials 2: Table 1

Table 1

Publication	Design	Disease	n	Prev. (%)	Sens. (%)	Spec. (%)	Tx A (%)	F/U (yrs.)	Stim.	PET /CT	CE-CT	US	Inclusion	Comment
Asa 2014 (17)	Retro	DTC	40	50	78.5	50		1.3	+/-	+	-	none	I-131-, hTg-, Tg-AB+	
Özdemir 2014 (16)	Retro	DTC	71	53.5	68.8	78.3		2	endo	+	-	all	hTg+, Tg-AB-, I-131-	
Giovanella 2013 (21)	Retro	DTC	102	48	94.2	94			none	+	-	all	hTg+, Tg-AB-, I-131-	
Ozkan 2013 (20)	Retro	PTC	59	76.3	80	27			+/-	+	-	some	hTg+ or Tg-AB+, I-131-	LSens 75 %<br>LSpec 70 %
Riemann 2013 (19)	Retro	DTC	327	40.7	92	95		1	+/-	+/-	-	all	hTg+ or US+ or susp.	4 centers
van Dijk 2013 (18)	Retro	DTC	52	25	69.2	92.3			+/-	+/-	-	some	After prim. Tx or rec.	
Bannas 2012 (27)	Retro	DTC	30	83.3	68	60			endo/rTSH	+	+	all	hTg+, I-131-	
Kunawudhi 2012 (26)	Pro	DTC	30	70	100	77.8		1	none	+	-	post	I-131-, hTg+	LSens 84 %<br>LSpec 72 %
Na 2012 (25)	Retro	PTC	68	66.2	69.4	66.7		2.8	endo	+	-	some	hTg+ or TgAB+, I-131-	
Prestwich 2012 (24)	Retro	DTC	47	55.3	69	76	29		rTSH	+	-	some	hTg+ or susp.	
Rosenbaum-Krumme 2012 (23)	Retro	DTC	90	12.2	96	94	12.2		endo	+	-	-	High-risk DTC at time of first I-131 ablation	
Vural 2012 (22)	Pro	DTC	104	76	87	77	39	3.8	endo	+/-	-	all	I-131-, US-, hTg+	
Kingpetch 2011 (29)	Retro	DTC	22	54.5	100	62.5		1	none	+	-	some	TgAB+, hTg-, I-131-	
Oh 2011 (28)	Retro	DTC	140	32.9	61	98		2.1	endo/rTSH	+/-	-	none	Susp. dMet or hTg+	Dist. met.<br>LSens 61 %<br>LSpec 97 %
Lal 2010 (34)	Retro	DTC	30	76.7	73.9	61.5	10	1.9	+/-	+	-	some	Susp.	
Piciu 2010 (33)	Retro	DTC	27	85.2	100	75	88.8		-	+	-	some	I-131-, US-, hTg+	
Razfar 2010 (32)	Retro	DTC	124	71	80.7	88.9	48.3	3.1	none	+	-	some		
Seo 2010 (31)	Retro	DTC	432	13.4	75.6	87		2	endo	+/-	-	all	All with FDG-PET/CT	
Vera 2010 (30)	Pro	DTC	44	43.2	95	95.8		2.6	rTSH	+	-	some	rTSH-stim. hTg > 2 µg/l, I-131	
Esteva 2009 (35)	Retro	DTC	50	78	82	64		1	endo	-		none	hTg+, I-131-	
Zuijdwijk 2008 (36)	Retro	DTC	39	76.9	92	88	51	2.8	none	+/-	-	none	hTg+ or susp..	

Publication	Design	Disease	n	Prev. (%)	Sens. (%)	Spec. (%)	Tx A (%)	F/U (yrs.)	Stim.	PET /CT	CE-CT	US	Inclusion	Comment
Freudenberg 2007 (38)	Retro	DTC	36	61.1	96	100	25	3	endo	+	+	all	I-131-,US-	LSens 99 %<br>LSpec 98 %
Mirallié 2007 (37)	Pro	DTC	45	84.4	61.5	0		0.5	endo	+	-	some	I-131-, hTg+	
Palmedo 2006 (39)	Pro	DTC	40	47.5	94.7	90.5	32.5	0	endo	+	-	some	I-131-	LSens 90 %<br>LSpec 91 %
Gabriel 2004 (40)	Pro	DTC	36	88.9	28	50	0	1	none	-	-	some	I-131-	
Hung 2003 (41)	Retro	PTC	20	95	89.5	100	0	1	endo	-	-	all	I-131-	
Frilling 2001 (44)	Pro	DTC	24	83.3	94.6	25	37.5	0	-	-	-	all	I-131-, hTg+	
Helal 2001 (43)	Pro	DTC	37	70.3	100	72.2	78.4	0.5	none	-	-	all	I-131-, hTg+	
Schlüter 2001 (42)	Retro	DTC	61	80.3	69.4	41.7	0	0	-	-	-	all	I-131-, hTg+	
Chung 1999 (47)	Retro	DTC	54	61.1	93.9	95.2	0	0	none	-	-	-	I-131-, susp.	
Grünwald 1999 (46)	Retro	DTC	222	49.1	75.2	90.2	0	0	+/-	-	-	some	susp..	
Wang 1999 (45)	Retro	DTC	37	54.1	70	76.5	51	0	+/-	-	-	-	I-131-	
Dietlein 1997 (48)	Pro	DTC	58	48.3	50	97.7	0	0	+/-	-	-	-	susp. or hTg+	
Feine 1996 (6)	Retro	DTC	41	82.9	83.4	100	0	0	endo	-	-	-		

Publications on the diagnostic performance of FDG-PET in patients with DTC and previous ablative therapy.

Design: retro = retrospective, Pro = prospective. Disease: TC = thyroid cancer, DTC differentiated TC, PTC = papillary TC, MTC = medullary TC. n = number of patients. Prev. = Disease prevalence. Sens. = Sensitivity. Spec. = Specificity. TxA = Beneficial alteration in therapy. F/U = mean or median follow-up. Stim. = mode of stimulation: endo = endogenous, + = endogenous or rTSH, var. = varying. PET/CT: + = PET-CT only, - = single-modality PET. CE-CT = contrast-enhanced CT performed as part of PET/CT examination. US = Ultrasound. Inclusion = Inclusion criteria: hTg+/- = detectable/undetectable hTg, I-131- = negative I-131 scintigraphy, rec. = manifest recurrence, susp. = suspected recurrence, Tg-AB+/- = positive/negative Tg antibodies. Comment: LSens/LSpec = Lesion-based sensitivity/specificity, dist. met. = distant metastases.
